# The regulation of health data sharing in Africa: a comparative study

**DOI:** 10.1093/jlb/lsad035

**Published:** 2024-01-18

**Authors:** Annelize G Nienaber McKay, Dirk Brand, Marietjie Botes, Nezerith Cengiz, Marno Swart

**Affiliations:** Division of Law, School of Business, Law & Social Sciences, Abertay University, Dundee, Scotland, United Kingdom of Britain; Department of Public Law, University of Pretoria, Pretoria, South Africa; School of Public Leadership, Stellenbosch University, Stellenbosch, South Africa; Division of Medical Ethics and Law, Faculty of Medicine and Health Sciences, Stellenbosch University, Stellenbosch, South Africa; Division of Medical Ethics and Law, Faculty of Medicine and Health Sciences, Stellenbosch University, Stellenbosch, South Africa; Faculty of Law, University of Cambridge, Cambridge, United Kingdom of Britain

**Keywords:** health data, data sharing, Africa, policy, comparative study, data governance

## Abstract

The sharing of health data is an essential component in the provision of healthcare, in medical research, and disease surveillance. Health data sharing is subject to regulatory frameworks that vary across jurisdictions. In Africa, numerous factors complicate the regulation of health data sharing, including technological, motivational, economic, and political barriers, as well as ethical and legal challenges. This comparative study examines the regulation of health data sharing in Africa by comparing and contrasting the legal and policy frameworks of five African countries. The study identifies gaps and inconsistencies in the current regulatory regimes and provides recommendations for improving the regulation of health data sharing in Africa.

## I. INTRODUCTION

The sharing of health data is crucial in improving healthcare outcomes, enhancing medical research, and ensuring effective disease surveillance, as demonstrated by the unprecedented sharing of health data by different countries and regions during the recent Covid-19 pandemic. Health data are pivotal for patient care, in developing new treatments for disease, monitoring disease outbreaks, and improving public health policies and interventions. Given the immense potential its application offers, it is not surprising that there is a growing interest in sharing health data in Africa and around the world.

Although the sharing of health data is a critical resource for enhancing the quality and efficiency of healthcare systems, it raises concerns about privacy, confidentiality, and data protection. These concerns are evident in an African context where the legal and regulatory frameworks governing data sharing are less well known and are considered less accessible than better-known frameworks such as the European General Data Protection Regulation (GDPR).^1^

In light of these concerns, the article presents a comparative study of the regulation of health data sharing in a selected number of countries in Africa. Our goal is to review and describe the data privacy and data export regulatory environment of these countries. By highlighting country-level strengths, best-practice, weaknesses, and gaps, we hope to inspire policy reform and stimulate debate about the need for regulatory reform, where necessary. The overarching aim of the study is to examine the legal and regulatory frameworks for health data sharing in selected African countries with the aim of identifying good practice as well as challenges in the regulation of health data sharing and to propose recommendations for improving the legal and regulatory frameworks for health data sharing governance in the region. We also aim to highlight country case studies from which lessons may be learned (especially by countries looking to reform their data policy and privacy regulatory frameworks).

The article is a companion or follow-up complementing the paper titled ‘Data sharing governance in sub-Saharan Africa during public health emergencies: Gaps and guidance’.^2^ The five countries selected for the comparative study are Ghana, Kenya, Nigeria, South Africa, and Uganda. These countries were chosen because they represent a range of ethical, legal, cultural, and technological contexts and all have made significant efforts to regulate health data sharing.

The article first provides an overview of the importance of health data sharing and the potential benefits and risks associated with such health data sharing. It then outlines the methodology used in the study, including justification of the selection of countries and the analysis of legal and regulatory frameworks for health data sharing. The study uses a comparative approach to analyse the legal and policy frameworks of these countries and focuses on issues such as data protection, consent, data ownership, and data-sharing agreements. The study considers several factors that influence the regulation of health data sharing in Africa. Lastly, we conclude with a discussion of the implications of the study for improving the regulation of health data sharing in Africa and propose recommendations for policymakers, research sponsors, researchers and other stakeholders in the region.

It is hoped that the findings of this study will provide valuable insight into the regulation of health data sharing in Africa and will inform the development of more effective regulatory frameworks, where necessary. It further is hoped that stakeholders will find our mapping exercise useful and practical. Researchers, specifically, need to know what is possible regarding data mining, data export, and other such activities. Research sponsors need to know where their research investment will yield the best global health benefit (for example, it could be considered a poor research investment in a setting where the country’s regulatory environment prohibits cross-border data sharing). Furthermore, African policymakers need to know and understand what their countries’ regulatory weaknesses are so that these may be addressed to attract greater research investment to the benefit of all. It is hoped that the study will contribute to the broader debate on the ethical, legal, and policy challenges in health data sharing and provide a basis for further research in this area.

## II. CONTEXT REGARDING HEALTH DATA SHARING

### II.A. What Is the Context of Health Data Sharing?

Enabled by various forms of wearables, surveillance and computing technologies, health data in particular are generated at an unprecedented speed and volume to the extent that new data management systems and tools have had to be developed to process and manage the complex integrated big data sets and the changes imposed on the ecosystem of health research, education, and medicine.^3^ Artificial intelligence (AI) enabled medical information systems and devices increasingly are used to identify potential relationships or patterns in medical data to gain useful knowledge for the purposes of improving diagnosis and treatment of patients, predict the spread and characteristics of infectious diseases, and assist in processing the challenges medical big data pose.^4^ In this context, big data is characterised according to the five ‘V’s: (1) volume, (2) velocity, (3) variety, (4) value, and (5) veracity.^5^

Specifically, medical data are characterised by disease diversity, heterogeneity of treatment and outcome, and the complexity of collecting, processing, and interpreting data.^6^ To produce big data sets, medical data are obtained from a variety of sources such as electronic health records, biometric data, patient report data, and clinical research data. The characteristics of medical *big* data differ from that of big data in other disciplines because collection, processing, and storage are much more difficult and costly due to these processes being subjected to various protocols, ethical approvals, and privacy preserving regulations. In addition, medical big data are not only large in scale but also are subjected to many and continuous updates or changes, are polymorphic in nature, and often incomplete and time sensitive.^7^

Consequently, the development of big data repositories in this context will lower collection costs, its aggregation may produce more complete and up-to-date data sets, allow for the production of richer research data sets to inform medical and health advancements, and increase global or continental cooperation to promote health research, therapeutic development, and clinical practice.^8^ To develop medical data repositories of real value, it is critical that institutions and countries, especially those sharing the same or similar geographies or epidemiologies that cause the same, similar, or shared health effects, are able effectively and efficiently to share their medical and health data. Unfortunately, a lack of knowledge about the different legal requirements relating to data sharing imposed by different countries in Africa poses an unnecessary obstacle in this regard. It is the aim of this paper to clarify these uncertainties in African countries with high levels of activity in heath research such as Ethiopia, Ghana, Kenya, Nigeria, South Africa, Tanzania, and Uganda.

### II.B. Africa’s Importance in a Clinical Trial Context

Africa has become an important destination for clinical research. This development is due to a combination of factors, including a large and diverse population; the high prevalence of diseases such as HIV, malaria, and tuberculosis; and the (relatively) low cost of conducting clinical and other research. In particular, sub-Saharan Africa is an attractive destination for clinical trials; countries such as South Africa, Kenya, and Nigeria lead the way in terms of the number of trials conducted.^9^ These trials have played a crucial role in advancing medical research; they have led to the development of new treatments and interventions to the benefit of many.

A reason Africa is an ideal setting for clinical trials is the high disease burden in the region. According to the World Health Organisation, 96 per cent of all malaria deaths occurred in Africa in 2020,^10^ and in 2021 over 70 per cent of all new HIV infections occurred in sub-Saharan Africa.^11^ This situation represents a unique opportunity for researchers to test new treatments in a population most affected by these diseases.

A further advantage of conducting clinical trials in Africa is the diversity of the African population.^12^ Africa is home to over 1.2 billion people,^13^ and there is considerable genetic diversity within and between countries.^14^ This factor presents an opportunity to study the effects of new treatments in a population that not only is representative of the global population but also possesses genetic diversity,^15^ a context particularly important for diseases such as cancer and cardiovascular disease where the genetic makeup of the patient can impact the effectiveness of the treatment.^16^

Lastly, the cost of conducting research in Africa sometimes is considerably lower than in countries such as the USA or those in Western Europe. Because research participants often are treatment naïve, there is no need for a prolonged ‘wash-out’ initial period to eliminate the effects of other medications, which makes for shorter clinical trials.^17^ Overall labor and facilities costs also tend to be lower in an African setting.^18^

### II.C. What Generally Are the Obstacles and Enablers with Regard to the Sharing of Health Data?

Data sharing is a widespread practice in business and government as well as in a research environment. It is a process of making available the same data to different people, organizations, or applications. In the context of modern information technology, such as a smartphone, there is an additional dimension to data sharing, namely, technology that enables fast and easy sharing of data between different applications and users. Data sharing, however, is not a new phenomenon and existed long before the development of laptops, smartphones, and artificial intelligence. Some examples are employers sharing employee data with medical aid and pension funds, and medical practitioners sharing the personal data of patients to enable appropriate diagnostics and care of patients. Effective data flows within and between countries are an essential element of a globalised world and data sharing takes place daily.

In recent years, and since the adoption of data protection legislation in many countries and the explosion of new information technology developments, there is a substantial interest in data sharing. This interest has resulted in data protection legislation that regulates the protection and lawful processing of personal data, for example, the GDPR in the European Union and the Protection of Personal Information Act 4 of 2013, in South Africa (POPIA).

Different terms are used in the different instruments and pieces of legislation to describe the person or organization that has primary responsibility for the lawful processing of personal information or data, for example, a responsible party (POPIA) or a controller (GDPR).

Although the term ‘data sharing’ commonly is used, in practice, it is not defined in data protection legislation such as the GDPR and POPIA. Despite the absence of a legal definition of data sharing, the legal basis for this practice needs to be clarified. The Information Commissioner (ICO) in the United Kingdom (UK) issued a Data Sharing Code of Practice (Data Sharing Code) in 2021 in terms of that country’s Data Protection Act (DPA), 2018^19^, which provides clear guidance regarding the practice of data sharing. In section 121 of the DPA, data sharing is described as ‘the disclosure of personal data by transmission, dissemination or otherwise making it available’. The Data Sharing Code deals with sharing data between controllers, i.e. the persons or institutions who decide on the reasons for the processing of personal data and how it should be done. Enabling access to the same data by different staff members in an organization is not regarded as data sharing since it is still the same organization that is processing the data. Although it is not legally mandated, the Code advises that it is good practice to have a data sharing agreement when data are to be shared between different operators. An agreement should clearly indicate the role of each party, the purpose of the data sharing, and what data will be shared. The legal basis for data sharing is provided by the rules applicable to lawful processing of personal data as described in the relevant data protection legislation, such as the Data Protection Act (DPA), 2018.^20^

Data sharing in healthcare as well as in health research is of critical importance to strengthen informed decision-making by health practitioners and to support innovation. The medical history of a patient (data subject) must be accessible to all the health practitioners and healthcare facilities attending to that patient in order to support informed diagnosis and appropriate treatment. Therefore, it is of the utmost importance that sharing of health data is properly regulated.

In the WHO policy on data sharing in health research, it is stated that public health can be advanced through^21^

‘appropriate sharing and reuse of health data, permitting analyses that:

(1) allow for the fullest possible understanding of health challenges;
(2) help develop new solutions; and
(3) ensure that decisions are based on the best available evidence.’

The variety of data sources used in the context of public health suggests relevant sharing of health data to respond effectively to health challenges. The increased use of digital technology, including artificial intelligence (AI), cloud computing, and application programming interfaces (APIs), creates opportunities for easier sharing of data between applications and users, which contributes to developing innovative health interventions.^22^ Sharing health data is inherent in the effective utilization of digital technology in health research as well as in the delivery of health care. Combining data from different sources, in particular using digital technology, has huge potential significantly to transform health care.^23^ Individualised patient care, also described as precision medicine, requires lots of data and the utilization of data from different sources that include data provided by the patient (e.g., health records, wearable digital devices, smartphone apps) as well as data obtained from other sources such as hospitals, industry partners, and academic research facilities. In order to ensure collaboration and optimal use of all the available data sources, data sharing needs to take place and must be within the applicable data protection legal framework.

In health research, it is quite common and often necessary that researchers from different institutions collaborate on a research project, which implies data sharing. It implies more than one responsible party (controller) who ensures compliance with the requirements for lawful processing of personal data. A data sharing agreement between the participating institutions is the appropriate way to ensure the joint commitment of the parties and a clear allocation of the responsibilities of each. Although non-personal data or anonymised data can be a part of a health research project, the legal protection of the data subject(s) always is central in the development of a data sharing agreement.

The WHO stipulates that data collection in health data programs under the auspices of the WHO should be done equitably, ethically, and efficiently and by adhering to the FAIR principles, which means that data should be

- Findable
- Accessible
- Interoperable, and
- Reusable.^24^

These principles imply some degree of standardization of data formats.

Data sharing in a health context often is a challenge, and these challenges can be categorised as follows:^25^

- Technical barriers, including incomplete data, and lack of interoperability of data;
- Motivational barriers, such as lack of incentives to share data;
- Economic barriers, such as the cost of processing and sharing data;
- Political barriers, such as policies that limit efficient data sharing practices;
- Legal challenges, including those relating to effective data security and data management, informed consent, and protection of privacy; and
- Ethical barriers, such as a lack of perceived reciprocity and proportionality, and insufficient interaction with data subjects about the use of their data.

Despite the challenges in effective data sharing, there are developments that function as enablers or strengthen data sharing in health care. The rapid development and use of new technology such as wearable devices, relevant APIs that facilitate the easy flow of data between applications, and AI facilitate effective data sharing. The use of big data in machine learning opens up new possibilities for health research, for example, through improved diagnostics in a variety of health conditions. Another enabling factor is the use of cloud storage, which is especially useful when researchers in various countries cooperate in a research project that involves large datasets. Although this section focuses on general data sharing in health care, the issues apply to data sharing when AI is used in health care.

## III. SELECTION OF COUNTRIES FOR COMPARATIVE REVIEW

### III.A. General

As stated above,^26^ the aim of the study is to compare the regulation of health data sharing in five African countries, South Africa, Ghana, Kenya, Nigeria, and Uganda, representing different regions. The study is a comparative desk-based review of the available and accessible regulatory instruments that focus on the legal and policy frameworks of the selected countries, including issues such as data protection, consent, data ownership, and data-sharing agreements. The study considers various factors that influence the regulation of health data sharing in Africa.

The five countries were selected based on several factors as outlined below, including more generally population size, geographic location, the availability of information on health data sharing regulation, and, importantly, their status as prime destinations for health research funding and internationally collaborative research.^27^

Specifically, the five countries were chosen based, first, on the number of their research outputs. In a ranking of the research output of African countries in terms of Health Information Management, the selected countries are ranked in the top 10 by Scopus for the period 1996 to 2022.^28^ The five countries are the focus of the companion paper to this contribution, as mentioned above.^29^

In addition, the five countries selected are ranked as receiving the most funding from major international funders of health research, namely, the US government, the EU, philanthropic organizations (such as the Gates Foundation), and international health agencies (such as WHO).^30^

South Africa was chosen due to its relatively advanced legal and regulatory framework for health data sharing, which encompasses the Constitution, 1996,^31^ the National Health Act 61 of 2003^32^, and the Protection of Personal Information Act 4 of 2013 (POPIA).^33^ South Africa is a foremost destination for internationally collaborative research^34^ and was the highest earner in terms of annual grant awards in 2020.^35^ Because of its relatively well-developed health infrastructure and relatively treatment-naïve population, unsurprisingly, South Africa is a sought-after destination for international collaborative research.^36^

Ghana, Kenya, Nigeria, and Uganda were selected as representing different regions and because they vary in terms of their legal and regulatory frameworks for health data sharing.^37^ They have a significant burden of disease and are considered priority areas for health research and development.^38^ They are prime destinations for health research funding from both public and private organizations; they received the following amounts in annual grant awards for health research in 2020: Ghana 10.85 million USD; Kenya 18.58 million USD; Nigeria 17.47 million USD; and Uganda 26.36 million USD, placing it second with regard to annual grant awards for health research in 2020.^39^

Data on the five countries’ regulation of health data sharing were collected, analysed, and collated into a table (Table 1). The data were compared across the five countries to identify similarities and differences in the legal and regulatory frameworks, technological infrastructure, and other factors affecting health data sharing.

**Table 1:** Comparative review of the regulation of data sharing in the selected countries

**GHANA**	
**Data protection and privacy laws**	Data Protection Act 843 of 2012
**Collection and processing conditions**	A person shall collect data directly from the data subject unless: the data is contained in a public record; the data subject has deliberately made the data public or has consented to the collection of the information from another source; the collection of the data from another source is unlikely to prejudice a legitimate interest of the data subject or is necessary for a number of expressly designated purposes; compliance would prejudice a lawful purpose for the collection. A data controller must ensure that the data subject is aware of: the nature of the data being collected; name and address of the person responsible for the collection; purpose for which the data is required for collection; whether or not the supply of the data by the data subject is discretionary or mandatory; consequences of failure to provide the data; authorised requirement for the collection of the information or the requirement by law for its collection; recipient of the data; nature or category of the data; existence of the right of access to and the right to request rectification of the data collected before collection Where collection is carried out by a third party on behalf of the data controller, the third party must ensure that the data subject has the information listed above.
**Cross-border transfers**	While no provisions in Act 843 specifically pertain to transfer outside of national borders, selling or offering to sell the personal data of another person anywhere constitutes an offense punishable by a fine of not more than two thousand five hundred penalty units, a term of imprisonment of not more than five years, or both. An advertisement that indicates that personal data is or may be for sale is an offer to sell the data.
**Authoritative body/ regulator/regulator**	The Data Protection Commission
**Penalties for non-compliance**	Offender is punishable by a fine (up to 250 penalty units) or imprisonment (up to two years), or both. Offenders of selling personal data are punishable by a fine (up to 2500 penalty units) or imprisonment (up to five years), or both. A penalty unit is equivalent to GHS 12.
**Cybercrime laws**	Electronic Communication Act 2008Act No. 775 Electronic Transactions Act 2008, Act No. 772
**Signatories of the African Union Convention on Cyber Security and Personal Data Protection**	Yes
**KENYA**	
**Data protection and privacy laws**	The Data Protection Act 2019
**Collection and processing conditions**	The processing of personal data must be: processed in accordance with the right to privacy of the data subject; processed lawfully, fairly and in a transparent manner in relation to any data subject; collected for explicit, specified and legitimate purposes and not further processed in a manner incompatible with those purposes; adequate, relevant, limited to what is necessary in relation to the purposes for which it is processed; collected only where a valid explanation is provided whenever information relating to family or private affairs is required; accurate and, where necessary, kept up to date, with every reasonable step being taken to ensure that any inaccurate personal data is erased or rectified without delay; kept in a form that identifies the data subjects for no longer than is necessary for the purposes which it was collected; not transferred outside Kenya, unless there is proof of adequate data protection safeguards or consent from the data subject.
**Cross-border transfers**	The Act prohibits cross-border data transfers unless such transfers are to a country with adequate levels of protection, the same as in Kenya, or approvals have been obtained after the data controller or data processor has given sufficient proof that measures have been put in place to protect the personal data.
**Authoritative body/regulator**	Office of the Data Protection Commissioner
**Penalties for non-compliance**	Offender is punishable by a fine (up to KES 5 million) or 1% of its annual turnover of the preceding financial year. Individuals will be liable to a fine (up to three million shillings) or imprisonment (up to 10 years), or both.
**Cybercrime laws**	Kenya Information and Communication Act 1998 The computer misuse and cybercrimes Act 2018
**Signatories of the African Union Convention on Cyber Security and Personal Data Protection**	No
**NIGERIA**	
**Data protection and privacy laws**	Nigerian Data Protection Act 2023
**Collection and processing conditions**	A data controller or data processor shall ensure that personal data is (a) processed, by such data controller or any data processor processing personal data on its behalf, fairly, lawfully, and in a transparent manner; (b) collected for specified, explicit, and legitimate purposes and not further processed in a way incompatible with those purposes; (c) adequate, relevant, and limited to the minimum necessary for the purposes for which the personal data was collected or further processed; (d) retained for no longer than is necessary to achieve the lawful bases for which the personal data were collected or further processed; (e) accurate, complete, not misleading and, where necessary, kept up to date having regard to the purposes for which the personal data were collected or are further processed; (f) processed in a manner that ensures appropriate security of the personal data, including protection against unauthorised or unlawful processing and access against loss, destruction, or damage and the data controller and data processor shall use appropriate technical and organizational measures to ensure the confidentiality, integrity, and availability of the personal data.
**Cross-border transfers**	The Act provides that personal data shall not be transferred from Nigeria to another country unless the recipient of the personal data is subject to a law, binding corporate rules, contractual clauses, code of conduct, or certification mechanism that affords an adequate level of protection with respect to the personal data in accordance with the Act, and upon the application of one of the laid down conditions in the Act.
**Authoritative body/regulator**	Nigeria Data Protection Commission
**Penalties for non-compliance**	Offender is punishable by a fine (higher maximum amount of N = 10,000,000 or standard maximum amount of N = 2,000,000) and 2% of its annual gross revenue derived from Nigeria in the preceding financial year.
**Cybercrime laws**	Cybercrime Act 2015
**Signatories of the African Union Convention on Cyber Security and Personal Data Protection**	No
**SOUTH AFRICA**	
**Data protection and privacy laws**	Protection of Personal Information Act 4 of 2013
**Collection and processing conditions**	There are eight conditions required for the lawful processing of personal information by or on behalf of a responsible party: 1. Accountability The responsible party must ensure that the conditions for lawful processing are satisfied. 2. Processing limitation Processing must be conducted lawfully, for necessary and not excessive purposes, in a manner that protects the legitimate interests of the data subject and does not infringe on their rights. Personal information may only be processed with the consent of the data subject (or competent person where the subject is a minor). Such consent is revocable at any time, and at such point, the responsible party must cease processing the information. Personal information may also be processed for a lawfully recognised purpose as specified in POPIA, such as the protection of a legitimate interest of the data subject. Generally, personal information must be obtained directly from the data subject unless an exception applies.
	3. Purpose specification Personal information must be collected for a specific, explicitly defined, lawful purpose related to a particular function or activity of the responsible party. In most circumstances, the responsible party must act to ensure the data subject is aware of this purpose. Personal information may not be retained for any longer than is necessary to achieve the purpose for which it was collected, barring certain exceptions. 4. Further processing limitation Further processing of personal information must be compatible with the original purpose for which it was collected, as determined by factors such as the nature of the information concerned, possible consequences of further processing on the data subject, the manner in which the information was collected, and contractual rights and obligations existing between parties. 5. Information quality The responsible party must take reasonably practicable measures to ensure that the personal information provided is accurate, complete, and not misleading. The purpose for which the personal information is collected or further processed determines what is reasonably practical under the circumstances. 6. Openness The responsible party must keep documentation of all processing operations and notify the data subject when collecting personal information, barring certain exceptions. 7. Security safeguards The responsible party is required to safeguard the integrity and confidentiality of personal information in its possession and/or under its control by taking the appropriate, reasonable technical and organizational measures to prevent loss, damage, or unauthorised destruction. Necessary measures are also to be taken to prevent unlawful access to or processing of personal information. 8. Data subject participation The responsible party must allow data subjects to exercise their rights under POPIA regarding their personal data.
**Cross-border transfers**	Prohibited, unless third-party data recipient is subject to law providing adequate data protection (substantially similar to POPIA); data subject consents; transfer is necessary for the performance of a contract between the responsible party and the data subject; transfer required for the conclusion of a contract in the interest of the data subject; transfer is to the benefit of the data subject, and it is not reasonably practical to obtain the subject’s consent, but subject would have reasonably given consent if it was possible.
**Authoritative body/regulator**	The Information Regulator
**Penalties for non-compliance**	Offender is punishable by a fine (up to R1,000,000) or imprisonment (up to 10 years), or both.
**Cybercrime laws**	Electronic Transactions and Communications Act 2002
**Signatories of the African Union Convention on Cyber Security and Personal Data Protection**	Yes
**UGANDA**	
**Data protection and privacy laws**	The Data Protection and Privacy Act 2019 The Data Protection and Privacy Regulations 2021
**Collection and processing conditions**	Prior consent of data subject needed for collection and processing of personal data, including medical data. Collection and processing of religious/philosophical beliefs, political opinion, sexual life, financial information, health status and medical records data are prohibited, unless: collected in terms of Uganda Bureau of Statistics Act; processed to perform a right or obligation imposed by law/employer; information is given freely with consent of data subject; processing is for legitimate purposes of organization or body. Collection must be for lawful, specifically defined purpose, related to activities of data controller. Before collection of data, data controller must provide data subject with information about data collection, control, and purpose; Only necessary or relevant data may be processed; Data collector/processor must ensure that data is complete, accurate, up to date, not misleading; Data subject may request correction of data and destruction; Further processing only allowed if it is compatible with purpose for which it was collected initially.
**Cross-border transfers**	Data processors are to ensure that the country in which data are processed or stored has adequate measures in place for the protection of personal data at least equivalent to the protection provided for by Uganda’s law. This processing is subject to the data subject’s consent. The provision checks on illegal transfers and abuse of data.
**Authoritative body/regulator**	The Personal Data Protection Office within the National Information Technology Authority
**Penalties for non-compliance**	Offender is punishable by a fine (up to UGX 4.8 million) or imprisonment (up to 10 years), or both.
**Cybercrime laws**	Computer Misuse Act 2011
**Signatories of the African Union Convention on Cyber Security and Personal Data Protection**	No

## IV. COMPARATIVE REVIEW OF THE REGULATION OF DATA SHARING IN THE SELECTED COUNTRIES

The countries selected for this comparative review, namely, South Africa, Ghana, Kenya, Nigeria, and Uganda, have enacted and enforceable data protection laws. Ghana imposed formal legislative data protection from October 2012, whereas Uganda (February 2019), Nigeria (June 2023), Kenya (November 2019), and South Africa (July 2020) enacted similar legislation only recently.

The countries have enforceable legislation that places an obligation to comply with the data processing requirements as set out in the respective acts on the appointed data processors and/or controllers and holds them accountable in case of harm suffered as a result of a data breach. Although different terms may be used, and the structuring of provisions differs, the laws in these countries limit personal data processing to a specific, lawful purpose based on consent obtained directly from the data subject, also providing for further or additional consent to be obtained if data are being processed for a purpose not specified during the initial stages of data collection. This requirement is in keeping with current international practice. Data subject participation is at the forefront in these laws; also, they provide for transparency and clear communication about the storage, security, access to, and ultimately the withdrawal or deletion (if requested by the data subject) of any data held by the processor or controller. In essence, these laws provide much the same data protection to the respective data subjects residing in each jurisdiction.

The enacted protections play a critical role when a decision about cross-border data transfers has to be made (for example, when research data are shared with another institution for analysis) and when additional safeguards and protections, such as Data Transfer Agreements (DTAs), must be negotiated and agreed upon before any cross-border data transfers take place. The cross-border transfer requirements of South Africa and Uganda closely resemble one another; both countries require the data-receiving country to have adequate measures in place for the protection of personal data that are equivalent to their provisions (South Africa or Uganda) and that the data subject consents to any cross-border transfer of their data. Again, it is in keeping with current international practice. In addition to these requirements, Kenya and Nigeria involve their local data authorities to the extent that the Data Protection Act in Kenya requires that proof must be provided to their Data Commissioner about the appropriate safeguards with respect to the security and the protection of personal data, including the safeguards available in the data-receiving jurisdiction. In Nigeria, the Data Protection Commission is to issue guidelines regarding the adequacy of the level of protection of personal data in another country, and may further make regulations to direct data controllers and data processors to inform it of the measures that are in place to substantiate the adequacy determination. The Commission may also determine if a country, region, or sector within a country provides an adequate level of protection (section 42 of the Nigeria Data Protection Act, 2023).

Ghana has not a specific provision for transfers of personal data outside of its national borders, which situation complicates any form of data exchange with Ghana because such interactions necessarily require protection supplementary to legislative protection in the form of DTAs. Consequently, where data protection laws in the respective data-providing and data-receiving countries are substantially similar and provide protection equal to the protection data enjoy in the data providing country, cross-border data transfers are legally permissible. If it is not the case, this legal *lacuna* may be bridged by using a detailed DTA to address issues not addressed in a specific country’s national legislative framework.

Although ‘health data’ is not explicitly defined in the **Nigerian** legislation, the definition of ‘sensitive personal data’ includes biometric data and the health status of a data subject. The processing of sensitive personal data, including health data, requires a ‘higher standard’ to be met and, as a result, the Nigeria Data Protection Act requires ‘explicit consent’ for the processing of sensitive personal data, as one of the legal grounds (section 30 of the Nigeria Data Protection Act).

The Data Protection Act in **Kenya** defines ‘health data’ as ‘data related to the state of physical or mental health of the data subject and include records regarding the past, present or future state of health, data collected in the course of registration for, or provision of health services, or data which associates the data subject which the provision of specific health services’. In this definition, it must be noted, a data controller can process personal data only when the data subject consents to the processing of the data ‘for one or more specified purposes’ or where the processing is ‘necessary’.^40^ Necessary processing, according to the DPA, is defined as processing for the ‘purpose of historical, statistical, journalistic, literature and art or scientific research’. Consequently, if processing for the purpose of scientific research is considered a lawful and ‘necessary’ ground for the processing of personal data, it seems the consent of the data subject may not be required.^41^ The requirements in respect of sensitive data are higher and sections 48–49 of the DPA [read with the Data Protection (General) Regulations of 2021] that regulate cross-border data transfers require the consent of the data subject with regard to the transfer of sensitive personal data, which includes health data, unless the transfer is in the ‘public interest’. In which case, the question—whether it is possible to transfer health data to be processed by another country for public health purposes or where it is considered in the ‘public interest’—applies to the situation in Kenya.

**Ghana’s** Data Protection Act encompasses the ‘physical, medical, mental health or mental condition or DNA of the data subject; and the sexual orientation of the data subject’ in the definition of special personal data.^42^ Medical data may be processed only in specific circumstances; processing for scientific purposes is exempt from the application of the Act, which means that consent by the data subject is not required. Personal data are defined as ‘data about an individual who can be identified (a) from the data, or (b) from the data or other information in the possession of, or likely to come into the possession of the data controller’ and does not explicitly include health data. However, health data also constitute identifiable data about an individual, complying with the definition of personal data outlined above. Health data processed for research purposes only is exempt from the provisions of the Act. As mentioned above, Ghana’s Data Protection Act does not contain specific provisions pertaining to transfer outside of its national borders, but if personal data is transferred from a data controller to a third-party data processor (who may or may not be outside the borders of Ghana), then: ‘1) the data controller must ensure that the data processor establishes and complies with security measures specified in the Act;^43^ 2) processing of the data must be governed by a written contract that determines the terms and conditions of the confidentiality and security measures necessary to ensure the integrity of the personal data;^44^ and 3) the data controller must ensure that the data processor outside of Ghana complies with the relevant laws of Ghana and must register with the Data Protection Commission’.^45^

Health data are not explicitly defined in **Uganda’s** Data Protection and Privacy Act but rather are included as part of the definition of ‘sensitive personal data’, that is, ‘personal data which relates to the religious or philosophical beliefs, political opinion, sexual life, financial information, *health status* or medical records of an individual’.^46^ Freely given, specific, informed, and unambiguous consent is required from every data subject before data may be collected or processed,^47^ except when collection and processing of personal data are for purposes of ‘national security’ or ‘for medical purposes’. Although health data are seen as a special type of personal data that requires consent before processing is legally allowed, health research is not a listed ground that qualifies for exemption from the general consent rule.^48^ Accordingly, health researchers have to rely on and show proof of consent from the data subject to process special personal data for research purposes. Section 19 requires consent from the data subject for the purpose of storing and processing data outside Uganda. This demand has an important implication for the cross-border transfer of data as consent will have to be in place before data may be transferred outside the country.

In terms of **South Africa’s** POPIA, health data are considered ‘special personal information’, which is defined as ‘personal information’ that includes ‘religious or philosophical beliefs, race or ethnic origin, trade union membership, political persuasion, *health* or sex life or biometric information of a data subject’. Health data may not be processed unless the data subject consents to processing for a specific purpose, unless processing is for historical, statistical, or research purposes to the extent that ‘i) the purpose serves a public interest and the processing is necessary for the purpose concerned; or ii) it appears to be impossible or would involve a disproportionate effort to ask for consent, and sufficient guarantees are provided for to ensure that the processing does not adversely affect the individual privacy of the data subject to a disproportionate extent’, in which case consent is not required.^49^ Section 72 of POPIA requires the data subject’s consent for transfers of personal information outside the Republic of South Africa.

## V. CONCLUSIONS DRAWN FROM COMPARATIVE REVIEW—OBSTACLES AND ENABLERS

The above paragraphs outline the nature of the protections afforded data subjects when their personal health data are processed and shared in the countries selected for the comparative review. Viewed from the perspective of the data subject, although these countries have enforceable legislation in place to protect the data subject from the inappropriate and/or illegal processing and sharing of their data, it is evident the focus, nature, and level of protection vary from country to country.

The following enablers and obstacles to maintaining an optimal data export regulatory environment in these countries have been identified.

### V.A. Enablers

The first enabler is the existence in the countries under review of an enforceable data processing framework in the form of dedicated legislation that sets out the requirements for legal and accountable data processing and sharing. The legislative frameworks of all the countries limit personal data processing to a specific, lawful purpose based on consent obtained directly from the data subject. This is an important enabler, as dedicated legislation not only protects the data subject from illegal or inappropriate data processing but also it gives direction to the sponsors of research as to their rights in relation to data gathered in research projects.

In all the countries surveyed, the responsibility for complying with data protection measures rests with the appointed data processors and/or controllers and, as pointed out above,^50^ they are held accountable in instances of harm suffered as a result of a data breach. The identification of specific persons who may be held accountable helps to ensure to escape liability these persons will take care to comply with data protection measures that have been enacted. Identification of a specific person as an appointed data processor and/or controller is the second enabler of accountable data processing.

Although different terms are used, and the structuring of provisions differs, the laws of these countries limit personal data processing to a specific, lawful purpose based on consent obtained from the data subject. The requirement of data subject consent is a third enabler identified in our study. Data subject consent is a requirement for the processing of personal information in the countries surveyed. Also, further or additional consent must be obtained in instances where the data are being processed for a purpose not specified during the initial stages of data collection. The consent requirement ensures not only data subject participation in data processing but also robust protection of the data subject. This requirement complies with current international standards regarding consent to data processing and sharing and is an important enabler of the sharing of health data, which is a critical resource for enhancing the quality and efficiency of healthcare systems.

A fourth enabler of accountable health data sharing is that the laws of the selected countries provide for transparency and clear communication about the storage, security, access to, and the withdrawal or deletion of any data held by the processor or controller. This fact safeguards the data subject’s privacy rights in instances of cross-border health data transfers. It is important to ensure these safeguards are enforced by the authorities. Transparency and clear communication must be an integral part of the consent process.

Furthermore, a fifth point, a benefit is that the countries under review have an institution or official in charge of overseeing that data processing requirements, laws, and regulations are complied with and to whom breaches may be reported for investigation. The fact this duty rests in a specific office or (in some cases) an independently appointed ‘watchdog’ increases the likelihood of compliance with data privacy laws and helps to ensure accountability. This official can ensure that data processing requirements as set out in the respective acts are complied with and, consequently, hold the appropriate actors accountable in case of harm suffered as a result of a data breach.

A sixth enabler of the accountable and legal sharing of health data that is identified is that in the countries under review, when processed personal information related to health is afforded special protection. This protection is significant as it shows an awareness of the heightened importance of data subjects’ privacy rights and also sensitivity to the consequences that flow from a data breach.

The seventh enabler is a requirement when decisions about the cross-border transfer of data have to be made (e.g. when health research data are shared with another institution for analysis), that there should be additional safeguards and protections in place such as DTAs in cases where the process of such transfer is not described comprehensively in legislation. These DTAs should be negotiated and agreed upon before any cross-border data transfers take place. Again, it is in keeping with current international best practices and enables the protection of data subjects and encourages ethical research practices.

### V.B. Obstacles

A significant obstacle in health data sharing is the lack of consistency in defining what is encompassed by the term ‘health data’ in the different countries’ legislation. This circumstance is an additional hurdle in cases of sharing health data across borders. Researchers and research sponsors must have certainty about which of their activities falls under the ambit of legislation that governs health data sharing for them to comply with legislation. It is urgent that an attempt is made in each regulatory process to define exactly what is understood by the term ‘health data’.

A second obstacle is that some countries’ legislation does not specifically provide for cross-border transfer of personal data. This circumstance creates an obstacle to the international sharing of data. Ghana has not enacted a provision that governs the transfer of personal health data outside its national borders. An absence of regulation, necessarily, complicates any form of data exchange with Ghana, as additional protections to supplement legislative protection in the form of DTAs will be required.

None of the countries under review exhibits this obstacle, but a third significant impairment to health data sharing is an instance where data processing is not subject to the informed consent of the data subject or where no process or guidance is in place for the data subject to provide further or additional consent if data is being processed for a purpose not specified during the initial stages of data collection. Data subject participation is necessary to ensure legitimacy and transparency in the process of data sharing. Measures to ensure data subject consent not only must be legislated they should be part of the actual day-to-day practice of those controlling and sharing health data.

Below, we turn to a few general observations and recommendations.

## VI. GENERAL OBSERVATIONS AND RECOMMENDATIONS

In conclusion, this comparative study aims to contribute to the growing body of literature on health data sharing in Africa. By comparing the legal and regulatory frameworks in South Africa, Ghana, Kenya, Nigeria, and Uganda, insight is gained into the obstacles and enablers in health data sharing in the region.

It is our intention that the findings of the study inform the development of robust regulatory frameworks that promote health data sharing while protecting individual privacy rights. Furthermore, it is hoped that the findings of our study serve to support the continued growth of health research funding in these countries. In highlighting country-level strengths, best-practice, weaknesses, and gaps, we hope to inspire policy reform and stimulate debate around a need for regulatory reform. Legal sharing of health data has been identified.

Regular interaction, communication, and sharing of best practices between the data protection authorities contribute to strengthening the regulatory frameworks for health data sharing in Africa, as well as helping these authorities to gain insight into the practical implementation of health data sharing in other countries, the better to protect data subjects.

The increasing use of new digital technology such as artificial intelligence in health care relies on the availability of substantial amounts of data, including personal data. It is necessary that the legal environment responds to these developments with a view to ensuring the protection of personal data and the responsible use of artificial intelligence in health care.

## Footnotes

^1^
European Parliament and of the Council of Europe of 27 April 2016, *Regulation (EU) 2016/679 on the Protection of Natural Persons with Regard to the Processing of Personal Data and on the Free Movement of Such Data, and Repealing Directive 95/46/EC (General Data Protection Regulation),*  https://eur-lex.europa.eu/legal-content/EN/TXT/PDF/?uri=CELEX:32016R0679 (accessed May 4, 2023).

^2^
Dirk Brand, Jerome A. Singh, Annelize G. McKay, et al., *Data Sharing Governance in Sub-Saharan Africa During Public Health Emergencies: Gaps and Guidance*, 118 S Afr J Sci, 1, (2022).

^3^
Cary J.R Schlick, Joshua P. Castle & David J. Bentrem, *Utilizing Big Data in Cancer Care*, 27 Surg Oncol Clin N Am, 641, (2018).

^4^
Blagoj Ristevski & Ming Chen, *Big Data Analytics in Medicine and Health Care*, 15, J Integr Bioinform, 1, (2018).

^5^
Sarah B. Scruggs, Karol Watson, Andrew I. Su, et al., *Harnessing the Heart of Big Data*, 116 Circ Res, 1115, (2015).

^6^
Ivo D. Dinov, *Methodological Challenges and Analytic Opportunities for Modeling and Interpreting Big Healthcare Data*, 5 Gigascience, 12, (2016).

^7^
United Nations Development Programme, *Human Development Report (2015)*, https://www.undp.org/publications/human-development-report-2015 (accessed May 4, 2023).

^8^
Akram Alyass, Michelle Turcotte & David Meyre, *From Big Data Analysis to Personalised Medicine for All: Challenges and Opportunities*, 33 BMC Med Genomics, 1, (2015).

^9^
See Clinical Trials Arena, *The Dynamics of Clinical Research in Africa: 2016–21*, https://www.clinicaltri  alsarena.com/comment/clinical-research-africa-2016-21/ (accessed May 4, 2023).

^10^
The World Health Organisation, *The Global Health Observatory – Malaria,*  https://www.who.int/data/gho /data/themes/malaria (accessed May 4, 2023).

^11^
The Joint United Nations Programme on HIV/AIDS (UNAIDS*), Global HIV & AIDS Statistics - Fact sheet*, https://www.unaids.org/en/resources/fact-sheet (accessed May 4, 2023).

^12^
See Lisa Shea, Jacqueline Pesa, Gabrielle Geonnotti, et al., *Improving Diversity in Study Participation: Patient Perspectives on Barriers, Racial Differences and the Role of Communities*, 25 Health Expect,197, (2022); Gaurav Puppalwar, Meenakshi Mourya, Ganesh Kadhe, et al., *Conducting Clinical Trials in Emerging Markets of Sub-Saharan Africa: Review of Guidelines and Resources for Foreign Sponsors*, 2015 J Clinical Trials, 23, (2015).

^13^
World Population Review, *Africa Population 2023*, https://worldpopulationreview.com/continents/africa-population (accessed May 4, 2023).

^14^
Michael C. Campbell & Sarah A. Tishkoff, *African Genetic Diversity: Implications for Human Demographic History, Modern Human Origins, and Complex Disease Mapping*, 9 Annu Rev Genomics Hum Genet, 403, (2008) and Vanessa Strüver, Sheraz Ali, Firas Fneish, et al., *Patient Benefit of Clinical Research in Diversely Advanced African Developing Countries,* 96 Curr Ther Res Clin Exp,1, (2022).

^15^
Shea, *supra* note 12.

^16^
Michèle Ramsay, Jantina de Vries, Himla Soodyall et al., *Ethical Issues in Genomic Research on the African Continent: Experiences and Challenges to Ethics Review Committees*, 8 Hum Genomics, 1, (2014); Michèle Ramsay, Nigel Crowther, Ernest Tambo, et al., *H3Africa AWI-Gen Collaborative Centre: A Resource to Study the Interplay Between Genomic and Environmental Risk Factors for Cardiometabolic Diseases in Four Sub-Saharan African Countries*, Glob Health Epidemiol Genom, 1, (2016).

^17^
See Strüver et al., *supra* note 14.

^18^

*Id.*

^19^
Information Commissioner’s Office, *Data sharing: a Code of Practice*, https://ico.org.uk/for-organisations/uk-gdpr-guidance-and-resources/data-sharing/data-sharing-a-code-of-practice/ (accessed May 4, 2023).

^20^
United Kingdom Parliament, *Data Protection Act 2018,*  https://www.legislation.gov.uk/ukpga/2018/12/ contents/enacted (accessed May 4, 2023).

^21^
The World Health Organisation, *Sharing and Reuse of Health-Related Data for Research Purposes: WHO Policy and Implementation Guidance*, https://www.who.int/publications/i/item/9789240044968 (accessed May 4, 2023).

^22^
Nina Schwalbe, Brian Wahl, Jingyi Song, et al., *Data Sharing and Global Public Health: Defining What We Mean by Data*, 2 Front Digit Health, 1, (2020).

^23^
National Academy of Medicine, *Sharing Health Data – The Why, The Will and The Way Forward*, https://nam.edu/wp-content/uploads/2022/01/Sharing-Health-Data-prepub-FINAL.pdf (accessed May 4, 2023).

^24^
World Health Organisation, *supra* note 21.

^25^
Schwalbe, *supra* note 22.

^26^
See para I above.

^27^
See World Health Organisation, *Investments on grants for biomedical research by funder, type of grant, health category and recipient*, https://www.who.int/observatories/global-observatory-on-health-research-and-development/monitoring/investments-on-grants-for-biomedical-research-by-funder-type-of-grant-health-category-and-recipient (accessed May 1, 2023).

^28^
See Scimago Journal & Country Rank, *Country rankings*, https://www.scimagojr.com/countryrank.php?region=Africa&category=3605 (accessed May 3, 21,023).

^29^
See *supra* note 2.

^30^
See World Health Organisation *supra* note 27.

^31^
Constitution of the Republic of South Africa, 1996, *Government Gazette* 17,678 of 18 December 1996.

^32^
Government Gazette 26,595 of 23 July 2004.

^33^
Government Gazette 37,067 of 26 November 2013.

^34^
World Health Organisation *supra* note 21.

^35^
US$123 89 million; see World Health Organisation *supra* note 27.

^36^
See para II above.

^37^
See below para IV.

^38^
See World Health Organisation *supra* note 21.

^39^

*Id.*

^40^
Kenya, DPA, sections 30(1)(a) and (b).

^41^
Kenya, DPA, section 30(1)(viii).

^42^
Ghana, DPA, section 96.

^43^
Ghana, DPA, section 30(1).

^44^
Ghana, DPA, section 30(2).

^45^
Ghana, DPA, section 30(4).

^46^
Uganda, DDPA, Section 9(1).

^47^
Uganda, DDPA, section 9(1).

^48^
Uganda, DPPA, sections 7(1) and (2).

^49^
South Africa, POPIA, section 27(2)(d).

^50^
See para IV above.

